# Isolation and transfection of myenteric neurons from mice for patch-clamp applications

**DOI:** 10.3389/fnmol.2022.1076187

**Published:** 2022-12-21

**Authors:** Samuel Kuehs, Laura Teege, Ann-Katrin Hellberg, Christina Stanke, Natja Haag, Ingo Kurth, Robert Blum, Carla Nau, Enrico Leipold

**Affiliations:** ^1^Department of Anesthesiology and Intensive Care, Center of Brain, Behavior and Metabolism (CBBM), University of Lübeck, Lübeck, Germany; ^2^Institute for Human Genetics and Genomic Medicine, Medical Faculty, Rhine-Westphalia Technical University of Aachen, Aachen, Germany; ^3^Institute of Physiology, Medical Faculty, Rhine-Westphalia Technical University of Aachen, Aachen, Germany; ^4^Department of Neurology, University Hospital of Würzburg, Würzburg, Germany

**Keywords:** single cell isolation, sodium channels, Na_V_1.9, patch-clamp, electrophysiology, myenteric neurons

## Abstract

The enteric nervous system (ENS) is a complex neuronal network organized in ganglionated plexuses that extend along the entire length of the gastrointestinal tract. Largely independent of the central nervous system, the ENS coordinates motility and peristalsis of the digestive tract, regulates secretion and absorption, and is involved in immunological processes. Electrophysiological methods such as the patch-clamp technique are particularly suitable to study the function of neurons as well as the biophysical parameters of the underlying ion channels under both physiological and pathophysiological conditions. However, application of the patch-clamp method to ENS neurons remained difficult because they are embedded in substantial tissue layers that limit access to and targeted manipulation of these cells. Here, we present a robust step-by-step protocol that involves isolation of ENS neurons from adult mice, culturing of the cells, their transfection with plasmid DNA, and subsequent electrophysiological characterization of individual neurons in current-clamp and voltage-clamp recordings. With this protocol, ENS neurons can be prepared, transfected, and electrophysiologically characterized within 72 h. Using isolated ENS neurons, we demonstrate the feasibility of the approach by functional overexpression of recombinant voltage-gated Na_V_1.9 mutant channels associated with hereditary sensory and autonomic neuropathy type 7 (HSAN-7), a disorder characterized by congenital analgesia and severe constipation that can require parenteral nutrition. Although our focus is on the electrophysiological evaluation of isolated ENS neurons, the presented methodology is also useful to analyze molecules other than sodium channels or to apply alternative downstream assays including calcium imaging, proteomic and nucleic acid approaches, or immunochemistry.

## 1 Introduction

The enteric nervous system (ENS) contains several hundred million neurons and thus represents the largest part of the autonomic nervous system. Largely independent of the central nervous system (CNS), the ENS regulates digestive functions such as peristalsis, fluid absorption and secretion, and detection of luminal stimuli (Grundy et al., 2006; Furness, 2012). The neurons of the ENS are organized in two major ganglionated plexuses. The myenteric plexus is located between the longitudinal and circular muscle layers of the gastrointestinal tract (GI tract), while the submucosal plexus is found between the circular muscle layer and the mucosa. The ganglia of both plexuses contain various neuron subtypes including motor neurons, interneurons, and intrinsic primary afferent neurons (IPANs) (Kunze et al., 1995; Furness et al., 1998, 2004). IPANs have a prominent role in the ENS. They sense physiological stimuli such as chemical signals, stretch and tension of muscles, and feed the information into local intramural reflex loops (Furness et al., 2004) that control effector structures including smooth muscle, blood vessels, mucosal glands, or epithelia.

Analyzing the functions of enteric neurons, particularly their electrophysiological profiles, is important to understand their involvement in enteric neuropathies and to evaluate therapeutic options aimed to modulate the electrical activity of the neurons. The patch-clamp technique is particularly suitable to study the excitability of neurons as well as the biophysical parameters of the underlying ion channel components. A model frequently used to characterize the electrophysiological properties of enteric neurons are wholemount preparations of the longitudinal muscle layer with attached myenteric plexus (LMMP: longitudinal muscle/myenteric plexus) (Kunze et al., 1995; Rugiero et al., 2002; Mao et al., 2006; Osorio et al., 2011). However, patch-clamp recordings from LMMP preparations are challenging because individual neurons must first be exposed using a combination of enzymatic treatment and local mechanical manipulation to gain access to the surface of neurons, which affects the success rate of experiments.

Here, we present a robust step-by-step protocol optimized to isolate, enrich, and cultivate neurons from the myenteric plexus of adult mice facilitating the electrophysiological analysis of the neurons in whole-cell patch-clamp experiments. The technique involves the preparation of LMMPs and their enzymatic digestion in two steps during which enteric neurons are enriched and separated from muscle tissue and debris. To demonstrate the viability of isolated neurons and their suitability for electrophysiological studies, we analyzed the action potential characteristics of the neurons and characterized the properties of endogenously expressed voltage-gated sodium channels (Na_V_ channels) in whole-cell current- and voltage-clamp experiments, respectively. In addition, we show that isolated myenteric neurons can be transfected with cDNA expression constructs by electroporation. As an application example, we overexpressed and electrophysiologically evaluated a mutant variant of human voltage-gated sodium channel subtype Na_V_1.9 (p.L396P) associated with hereditary sensory and autonomic neuropathy type 7 (HSAN-7) (King et al., 2017), a complex disorder affecting both peripheral nociceptive neurons and enteric neurons, leading to congenital analgesia and severe gastrointestinal dysfunction (Leipold et al., 2013; Phatarakijnirund et al., 2016; Huang et al., 2017; King et al., 2017).

Even though the presented methodology has been optimized to use the neurons in electrophysiological assays, neurons generated with these methods are also compatible with other cell biology applications such as calcium imaging, proteomic and nucleic acid methods, or immunochemistry. In addition, transient transfection of isolated enteric neurons with cDNA expression constructs is easy to perform, which is expected to facilitate systematic overexpression studies with these cells.

## 2 Materials and equipment

### 2.1 Methods

#### 2.1.1 Animals

Male and female C57BL/6JRj wild type mice at an age of 8–12 weeks were used in this study. Mice were housed in individually ventilated Green Line cages (Techniplast, Hohenpeißenberg, Germany) under a 12-h light-dark cycle and fed an autoclaved pelleted mouse diet *ad libitum*. Animals were euthanized by isoflurane inhalation following cervical dislocation according to protocols approved by the local animal welfare committee (27_2018–11–13). Animal care and experimental procedures were performed in full accordance with the German Animal Welfare Act and all corresponding regulations.

#### 2.1.2 Immunochemistry

Longitudinal muscle/myenteric plexus pieces were obtained from the small intestine of adult mice as detailed in steps 3.3–1 to 15 of the protocol [Section “3.3 Dissection and preparation of LMMP from mice (45–60 min),” this study] and pinned with stainless steel needles to silicone elastomer-coated dishes filled with ice-cold complete saline solution (CSS, Section “3.1 Setup of reagents and tools,” this study). To fix the tissue, CSS was replaced with phosphate-buffered saline (PBS) containing 4% paraformaldehyde (PFA) (Roth, Karlsruhe, Germany, 73981), and samples were incubated for 4 h at RT. Subsequently, samples were washed three times with PBS and stored at 4°C for up to 5 days. Immediately before exposure to primary antibodies, samples were washed three times for 10 min each in PBS. Afterward, the tissue was permeabilized and blocked for 1.5 h at RT with blocking buffer consisting of PBS supplemented with 0.1% Triton X-100 (Sigma-Aldrich Chemie GmbH, Taufkrichen, Germany, T9284), 0.1% Tween 20 (Sigma-Aldrich Chemie GmbH, Taufkrichen, Germany, P9416) and 10% Normal Donkey Serum (NDS) (Biozol, SBA-0030-01). Next, primary antibodies were diluted in blocking buffer (anti-βIII Tubulin, Biolegend Cat# MMS-435P, RRID:AB_2313773 at 1:500; anti-CGRP Gt, Abcam Cat# ab36001, RRID:AB_725807 at 1:500) and added to the tissue for overnight incubation at 4°C. The following day, samples were washed three times for 15 min each with wash buffer consisting of PBS containing 0.1% Triton X-100 and 0.1% Tween 20 before they were incubated with secondary antibodies diluted in blocking buffer (Donkey anti-goat H+L labeled with Alexa Fluor 647, Thermo Fisher Scientific, Waltham, MA, USA, Cat# A21447, RRID:AB_2535864 at 1:400; Donkey anti-mouse H+L labeled with Alexa Fluor 488, Thermo Fisher Scientific Cat# A21202, RRID:AB_141607 at 1:400) for 1.5 h at RT in the dark. Subsequently, samples were washed three times for 15 min each time with wash buffer followed by 10 min incubation with DAPI solution (Sigma-Aldrich Chemie GmbH, Taufkrichen, Germany, MBD0015 at 1:1000). Samples were then washed twice in the dark with PBS for 10 min each and then rinsed once with ddH_2_O (double distilled water) before being mounted on microscopic slides using Prolong Gold mounting medium (Thermo Fisher Scientific, Waltham, MA, USA, P10144).

For immunocytochemistry of myenteric neuron cultures, the protocol was modified as follows. Myenteric neuron cultures were grown on glass coverslips for 72 h before being washed three times with PBS for 5 min each time. Cells were permeabilized by incubation for 10 min in PBS containing 0.3% Triton X-100 and then blocked for 30 min with blocking buffer consisting of PBS containing 0.1% Triton X-100 and 10% NDS. Cells were incubated overnight at 4°C with the primary antibody (anti-βIII Tubulin, Biolegend Cat# MMS-435P, RRID:AB_2313773) diluted 1:500 in blocking buffer. After washing with PBS for 5 min three times, samples were incubated for 1.5 h in the dark at RT with the secondary antibody (Donkey anti-mouse H + L labeled with Alexa Fluor 488, Thermo Fisher Scientific Cat# A21202, RRID:AB_141607) diluted 1:400 in blocking buffer. Cells were then washed in PBS for 5 min before they were incubated with DAPI solution for additional 5 min. Finally, cells were washed three times with PBS followed by a washing step with ddH_2_O and mounted on microscopic slides using Prolong Gold mounting medium.

Fluorescence images were acquired with a Leica TCS SP5 confocal microscope controlled by LAS AF software (both Leica Microsystems, Wetzlar, Germany). Contrast and brightness of the images were adjusted using Fiji software (Schindelin et al., 2012), no other image processing steps were performed.

#### 2.1.3 Viability assay

To assess viability of isolated myenteric cells, a two-color live-dead assay using fluorescein diacetate (FDA, F7378, Sigma-Aldrich) and propidium iodide (PI, 537059, Sigma-Aldrich) was performed (Jones and Senft, 1985). In this assay, FDA serves as a viability probe. After entering cells, the non-fluorescent FDA is rapidly converted by cytosolic esterases to the green fluorescent fluorescein, indicating viable cells. In contrast, PI is cell impermeant and generally excluded from viable cells. It can pass exclusively through disordered membranes of dead cells, where it forms a red fluorescent complex with double-stranded DNA. Briefly, separate stock solutions of FDA and PI were prepared by dissolving 5 mg of FDA in 1 ml acetone and 2 mg PI in 1 ml sterile PBS and stored at −20 and 4°C, respectively, until use. Staining medium was prepared freshly on the day of preparation by combining 5 ml FCS-free DMEM/F12 (1:1) with 8 μl of FDA stock solution and 50 μl of PI stock solution. Immediately after completing isolation of myenteric neurons [Section “3.4 Digestion of LMMPs and enrichment of ganglia (timing 6.5 h),” step 3.4–11, this study], cells were pelleted by centrifugation for 2 min at 250 × *g* and resuspended in 500 μl staining medium. Subsequently, 50 μl-aliquots of the cell suspension were seeded to separate wells of a 24-well culture plate followed by addition of 450 μl FCS-free DMEM/F12 (1:1). After allowing the cells to settle for 10 min, cells were imaged using an Axiovert A1 fluorescence microscope (Carl Zeiss Microscopy Deutschland GmbH, Oberkochen, Germany) equipped with a pE-300 LED light source (CoolLED Ltd., Andover, United Kingdom) and a Gryphax Wega CMOS camera operated by Gryphax software (both JENOPTIK Optical Systems GmbH, Jena, Germany). Final images of FDA and PI channels were analyzed using CellC software (Selinummi et al., 2005) to ensure unbiased counting of live and dead cells.

#### 2.1.4 Plasmid DNA

The cDNA expression constructs used for transfection of myenteric neurons contained the coding sequence of either human Na_V_1.9 or human Na_V_1.9-L396P under the control of the CMV promoter and a separate reporter cassette encoding the enhanced green fluorescent protein (eGFP) controlled by the SV40 promoter. Transfection-grade plasmid samples were purified from *E. coli* cells (Strain: JM109, L2005, Promega GmbH, Walldorf, Germany) using the NucleoBond Xtra Midi EF kit (Cat# 740412.50, Macherey-Nagel GmbH & Co. KG, Düren, Germany) according to the manufacturer’s instructions. Transfection of isolated myenteric neurons with these constructs is described in detail in steps 3.5–1 to 8 of the protocol [Section “3.5 Transfection of myenteric neurons with plasmid DNA (timing: 20 min),” this study].

#### 2.1.5 Electrophysiology

Voltage- and current-clamp recordings were performed in the whole-cell configuration of the patch-clamp method using an EPC10 patch-clamp amplifier operated by PatchMaster software (HEKA Elektronik, Lambrecht, Germany). Recordings were performed at a constant temperature of 20 ± 0.5°C using a microincubation stage (ALA Scientific Instruments, Farmingdale, NY, United States) feedback-controlled by a PTC-10 temperature controller (NPI Electronic GmbH, Tamm, Germany). Patch pipettes were pulled from borosilicate glass capillaries and coated with silicone elastomer (RTV 615, Momentive Performance Materials, Waterford, NY, United States) to reduce tip capacitance. Series resistance was corrected electronically up to 80%. All voltages were corrected off-line for the liquid junction potential (–7 mV). Bath solution for voltage-clamp recordings contained (in mM) 130 NaCl, 2 KCl, 2.5 CaCl_2_, 1 MgCl_2_, 10 HEPES (pH 7.4. with NaOH) and was supplemented with 20 TEA–Cl, 1 4-AP to suppress the activity of potassium channels endogenously expressed in myenteric neurons. In addition, 0.1 mM CdCl_2_ was added to the bath to block voltage-gated calcium channels (Lansman et al., 1986; Yang et al., 1993) further facilitating the isolation of Na_V_-mediated currents. Where indicated, the bath solution was further supplemented with 1 μM Tetrodotoxin (TTX) (Alomone Labs, Jerusalem, Israel) to block endogenous TTX-sensitive Na_V_ channels. The corresponding pipette solution contained (in mM) 10 NaCl, 130 CsF, 10 EGTA, 10 HEPES (pH 7.4 with CsOH). Bath solution for current-clamp recordings contained (in mM) 120 NaCl, 3 KCl, 2.5 CaCl_2_, 1 MgCl_2_, 30 HEPES, 15 glucose (pH 7.4 with NaOH), and the pipette 125 KCl, 8 NaCl, 1 CaCl_2_, 1 MgCl_2_, 0.4 Na_2_-GTP, 4 Mg-ATP, 10 EGTA, 10 HEPES (pH 7.3 with KOH). Data were analyzed with FitMaster (HEKA Elektronik) and IgorPro (WaveMetrics, Lake Oswego, OR, USA) software. Data are presented as means ± s.e.m., (*n*) with *n* being the number of independent experiments. Statistical comparisons of two groups of data were made using the two-tailed Student’s *t*-test when appropriate.

##### 2.1.5.1 Voltage clamp recordings

For voltage-clamp recordings, the holding potential was set to −137 mV. Data were low-pass filtered at 3 kHz and sampled at an interval of 40 μs. Leak and capacitive currents were recorded using a P/6 protocol with six leak pulses, each with 0.15 times the amplitude of the test pulse P. Summed and scaled leak traces were subtracted online using the leak pulse feature available in PatchMaster software.

Activation of Na_V_ channels was measured with test depolarizations ranging from −127 to 23 mV applied in steps of 10 mV every 10 s. The voltage dependence of channel activation was estimated from peak current densities using the following formalism:


(1) $$\frac{I\,\left(V\right)}{C_{m}}\,\Gamma \cdot \left(V-E_{r\,e\,v}\right)\cdot \frac{1}{1+e^{-\frac{\left(V-V_{m}\right)}{k_{m}}}}$$

with the cell membrane capacitance *C*_m_, conductance density Γ, and the reversal potential (*E*_rev_). *V*_m_ is the half-maximal activation voltage and *k*_m_ the corresponding slope factor.

To measure steady-state inactivation, Na_V_ channels were activated with a first 50 ms test pulse to −37 mV followed by a conditioning interval of 500 ms at voltages ranging from –147 to –7 mV in increments of 10 mV. Peak currents of not inactivated channels were measured in a subsequent 50 ms test pulse to −37 mV. The repetition interval was 10 s. The current amplitude after conditioning (*I*_500_) normalized to the control current amplitude before conditioning (*I*_0_) was described by the following Boltzmann formalism:


$$\frac{I_{500}}{I_{0}}=h_{m\,a\,x}-\left(h_{m\,a\,x}-h_{m\,i\,n}\right)\cdot$$

(2) $$\left(\frac{1-P}{1+e^{-\left(V-V_{h\,f}\right)/k_{h\,f}}}+\frac{P}{1+e^{-\left(V-V_{h\,s}\right)/k_{h\,s}}}\right)$$

with *h*_min_ and *h*_max_ being the minimal and maximal channel availability, and the half-maximal inactivation voltages *V*_hf_ and *V*_hs_ characterizing the inactivation of fast and slow inactivating current components, respectively; *k*_hf_ and *k*_hs_ are the corresponding slope factors. In experiments where either fast or slow inactivating current components were evident, *P* was set to 0 or 1, respectively.

##### 2.1.5.2 Current clamp recordings

Cells were first clamped to a holding potential of −67 mV in the whole-cell voltage-clamp recording mode, and then Na_V_-mediated inward currents were recorded with a 50 ms test depolarization to −17 mV before proceeding to the current-clamp recording mode. The resting membrane potential (RMP) of not spontaneously firing cells was measured in current-clamp mode by zero current injection. Subsequently, single action potentials were evoked by injecting a current of 50–100 pA for a period of 10 ms, followed by a 400-ms period without current injection. Parameters characterizing individual action potentials, including action potential peak voltage (*V*_peak_), minimum after-hyperpolarization voltage (*V*_AHP_), the voltage threshold of action potential firing (*V*_th_), and action potential width at threshold voltage (*Width*_th_), were analyzed with IgorPro software using customized scripts. *V*_th_ was defined as voltage at which d*V*/d*t* reached the level of 0.03x (d*V*/d*t*_max_ − d*V*/d*t*_min_) + d*V*/d*t*_min_ (Leipold et al., 2013). For all experiments, the threshold for action potential detection was set to 0 mV.

Trains of action potentials were evoked repetitively by 500 ms current injections, ranging from −20 to 90 pA in increments of 10 pA. The sampling interval was 20 μs for all current-clamp recordings. Spontaneously firing cells were excluded from the analysis of RMP.

Membrane input resistance (*R*_in_) was determined by the slope of linear fits to hyperpolarizing voltage responses to current injections ranging from −20 to 0 pA in steps of 10 pA.

## 3 Procedure

### 3.1 Setup of reagents and tools

All materials and equipment required for the isolation and transfection of myenteric neurons from mice are listed in Table 1.

**TABLE 1:** Materials and equipment for isolation and transfection of myenteric neurons.

Item	Supplier (Order number)
**Animals**	
C57BL/6JRj mice	Janvier Labs
**Reagents**	
Accutase™	Sigma-Aldrich Chemie GmbH, Taufkirchen, Germany (A6964)
Boric acid	Sigma-Aldrich Chemie GmbH, Taufkirchen, Germany (B6768)
Bovine serum albumin (BSA)	Sigma-Aldrich Chemie GmbH, Taufkirchen, Germany (A2153)
DNAse I	Thermo Fisher Scientific, Waltham, MA, USA (EN0521)
Dulbecco’s Modified Eagles Medium with Ham’s F12 (DMEM/F12 1:1)	Pan-Biotech GmbH, Aidenbach, Germany (P04-41154)
Fetal calf serum (FCS)	Sigma-Aldrich Chemie GmbH, Taufkirchen, Germany (F7524)
Isoflurane™	Baxter International, Deerfield, IL, USA (HDG9623)
Liberase™ TH	F. Hoffmann-La Roche, Basel, Switzerland (05401135001)
Liberase TM	F. Hoffmann-La Roche, Basel, Switzerland (5401127001)
Penicillin/Streptomycin	Biochrom GmbH, Berlin, Germany (A2213)
Phosphate-buffered saline (PBS)	Biowest, Nuaillé, France (L0615-500)
Poly-L-Lysine	Sigma-Aldrich Chemie GmbH, Taufkirchen, Germany (P9155)
Roswell Park Memorial Institute (RPMI) medium	Gibco™ by Thermo Fisher Scientific, Waltham, MA, USA (52400-017)
Trypsin inhibitor	F. Hoffmann-La Roche, Basel, Switzerland (10109886001)
**General materials, tools, kits**	
Angled forceps	Aesculap, Tuttlingen, Germany (BD313R)
Straight micro forceps	Aesculap, Tuttlingen, Germany (BD331R)
Spring scissors	Nopa instruments, Tuttlingen, Germany (AC 776/01)
Surgical scissors	Aesculap, Tuttlingen, Germany (BC110R)
Micropipettes (200 and 1,000 μl) with disposable tips	Eppendorf SE, Hamburg, Germany (3123000055, 3123000063)
Sterile glass Pasteur pipettes	Th. Geyer, Renningen, Germany (7691060)
Cotton swabs (Q-tips)	NOBA Verbandmittel, Wetter, Germany (974116)
Cell culture dishes (90 mm diameter)	Th. Geyer, Renningen, Germany (7696773)
Cell culture dishes (35 mm diameter)	Greiner Bio-One GmbH, Frickenhausen, Germany (627161)
Glass beakers (100 ml)	Th. Geyer, Renningen, Germany (7607553, 6052080)
Conical centrifuge tubes (15 and 50 ml)	Th. Geyer, Renningen, Germany (62.554.009, 62.547.254)
Microcentrifuge tubes (1.5 ml)	Sarstedt, Nümbrecht, Germany
Round glass coverslips (12 mm diameter)	Thermo Fisher Scientific, Waltham, MA, USA (0880)
24-Well cell culture plate	Greiner Bio-One GmbH, Frickenhausen, Germany (662160)
P3 Primary cell 4D-Nucleofector™X Kit S	Lonza Group, Basel, Switzerland (V4XP-3032)
**Devices**	
Dry bath for microcentrifuge tubes	Hangzhou Bioer Technology Co., Hangzhou, China (CHB-202)
Micro-centrifuge	Neuation Technologies, Gandhinagar, India (iFuge M12P)
Cooling centrifuge	Hettich GmbH & Co. KG, Tuttlingen, Germany (320-R)
Nutating shaker (Nutator), alternatively a rocking shaker	Biozym Scientific GmbH, Hessisch Oldendorf, Germany (55H3D1020-E)
Stereo microscope	Carl Zeiss, Oberkochen, Germany (Stemi-2000)
Cold light source with flexible light guides	Schott AG, Mainz, Germany (KL300LED)
Incubator (95% O_2_−5% CO_2_)	Eppendorf, Hamburg, Germany (Galaxy 170 S)
4D-Nucleofector™ (Core Unit, X Unit)	Lonza Group, Basel, Switzerland

**Fire-polished Pasteur pipettes:** Expose the tip of a glass Pasteur pipette to a Bunsen flame while rotating the pipette around its longitudinal axis until its edges are polished. Fire-polish Pasteur pipettes in batches of 10–15 and store them in a sterile box until use.

Prepare the following buffers and media in advance:

**Poly-L-Lysine coating solution:** Dissolve 3.09 g boric acid and 0.1 g NaOH in 400 ml ddH_2_O, adjust the pH to 8.5 and fill up with ddH_2_O to a final volume of 500 ml. Add 50 mg of Poly-L-Lysine and store aliquots of 5 ml at −20°C until use.

**Liberase Blendzyme aliquots:** Reconstitute Liberase Blendzymes TH and TM in sterile PBS to a final concentration of 60 and 10 U/ml, respectively. Store aliquots of 50 μl at −20°C until use.

**Complete saline solution (CSS):** Complete saline solution (Dib-Hajj et al., 2009) comprises 137 mM NaCl, 5.3 mM KCl, 1 mM MgCl_2_, 3 mM CaCl_2_, 25 mM Sorbitol, 10 mM HEPES in ddH_2_O. Adjust the pH to 7.2 with NaOH, filter-sterilize, and store at 4°C until use for up to 4 weeks. It is recommended to prepare CSS in batches of 1 L. For each experimental animal, it is recommended to prepare 500 ml of CSS and to oxygenate it for at least 15 min before starting the preparation.

**Culture medium:** The culture medium is composed of 89.5% (vol/vol) DMEM/F12 (1:1), 9.5% (vol/vol) fetal calf serum (FCS), and 1% (vol/vol) Penicillin/Streptomycin.

**Trituration medium:** Add 5 ml DMEM/F12 containing 1% (vol/vol) Penicillin/Streptomycin to a 15 ml conical centrifuge tube and supplement with 7.5 mg BSA and 7.5 mg trypsin inhibitor (TI). Trituration medium can be stored at 4°C for up to 2 weeks.

The following media must be prepared fresh on the day of the experimentation (**timing: 30 min**):

**Digestion medium A:** Add 3 U of Liberase Blendzyme TH (dissolved in 50 μl PBS) and 10 μl DNAse I solution (1 U/μl) to a 1.5 ml microcentrifuge tube and fill up to 1.2 ml with FCS-free DMEM/F12 containing 1% (vol/vol) Penicillin/Streptomycin. Keep the solution on ice.

**Digestion medium B:** Combine 1.15 ml of Accutase™ with 0.5 U of Liberase Blendzyme TM (dissolved in 50 μl PBS) in a 1.5 ml microcentrifuge tube and add 15 μl of a 50 mM EDTA solution. Keep the solution on ice.

**RPMI medium:** Add 1 ml of RPMI medium containing 1% (vol/vol) Penicillin/Streptomycin to a 1.5 ml microcentrifuge tube and store it on ice. **Note:** RPMI medium is only required if isolated myenteric neurons are to be transfected before seeding.

### 3.2 Preparation of glass coverslips (timing: 90 min)

**Note:** All steps involved in the preparation of glass coverslips should be performed under sterile conditions. Glass coverslips should be autoclaved in advance. Although it is sufficient to coat coverslips about 2 h before seeding cells, it is recommended to prepare them at least one day before the intended use. Typically, six coverslips are sufficient for seeding enteric neurons obtained from the small intestine of one mouse.

1. Rinse autoclaved glass coverslips (12 mm in diameter) with 95% EtOH to remove any particles.
2. For each animal to be used, place 6 coverslips into separate wells of a 24-well cell culture plate. Let them air-dry.
3. Add 400 μl of Poly-L-Lysine coating solution to each coverslip and incubate them at RT for 30 min to 1 h.
4. Remove the Poly-L-Lysine coating solution.
5. Wash three times with sterile PBS and allow coverslips to dry completely.
6. Sterilize coated coverslips with UV light for 10 min in a laminar flow box.

**Comment:** If not needed the same day, coated and sterilized coverslips can be stored for up to 2 weeks at 4°C.

**FIGURE 1:**
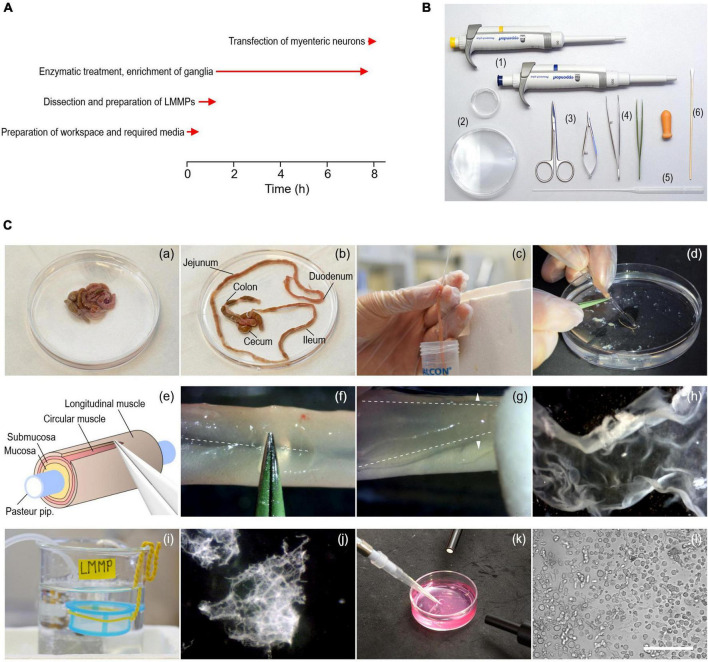
Procedure to isolate myenteric neurons from the gastrointestinal tract of adult mice. **(A)** Timing (red arrows) of experimental steps involved in the isolation and transfection of murine myenteric neurons. **(B)** Tools required include 1–ml and 200–μl pipettes with corresponding tips (1), 35- and 90-mm culture dishes (2), a pair of scissors of different sizes (3), coarse and fine tweezers (4), fire-polished Pasteur pipettes (5), and cotton swabs (6). **(C)** Series of pictures covering different steps of the isolation procedure. (a,b) Intestine from an animal before (a) and after (b) removal of the mesentery. (c) Pieces of the small intestine (duodenum, jejunum, ileum) are cleaned from content using a fire-polished Pasteur pipette. (d) A piece of intestine slid onto the thin end of a fire-polished Pasteur pipette. (e) Cartoon illustrating the extraction of LMMPs from tissue pieces. (f) The longitudinal muscle layer is carefully scratched along the mesenterial line (dashed line) using forceps. (g) The longitudinal muscle layer with the myenteric plexus attached (LMMP) is gently peeled off with a wettened cotton swab using light strokes applied perpendicular to the longitudinal axis (arrowheads). (h) Isolated piece of LMMP. (i) A cell strainer installed in a glass beaker serves as a storage reservoir for isolated LMMP pieces. (j) Pieces of LMMP after enzymatic digestion for 5 h. The ganglionated network of the myenteric plexus becomes visible. (k) Harvesting of myenteric plexus pieces as shown in panel (j) with a pipette under microscopic control. (l) Brightfield image showing a mixed cell population enriched with myenteric neurons, imaged directly after seeding the cells on glass coverslips. Scale bar: 100 μm.

7. On the day of the experiment: Before starting the isolation procedure (Figure 1A), add 1 ml of culture medium to each coverslip and incubate them at 37°C in a 95% O_2_ − 5% CO_2_ incubator until cell seeding.

### 3.3 Dissection and preparation of LMMP from mice (45–60 min)

Prepare the surgery table and place all tools required (Figure 1B) next to the surgery area.

**Important:** Clean the work area before starting the isolation procedure, use sterile instruments and wear gloves to minimize the risk of contamination.

1. Prepare fresh aliquots of digestion media A and B in 1.5 ml microcentrifuge tubes as described above and place the tubes on ice.
2. Place CSS on ice and bubble with carbogen for at least 15 min.
3. Place three 100 ml glass beakers on ice and fill each with approximately 50 ml of ice-cold CSS. Label beakers with “Dirty,” “Clean,” and “LMMP,” and continuously bubble the LMMP beaker with carbogen. Wrap one end of a piece of insulated wire (approx. 20 cm length) around a cell strainer (mesh size 300 μm) and bend the other end of the wire to form a hook (Figure 1C-i). Place the cell strainer, which will serve as storage reservoir for LMMP pieces, in the LMMP beaker so that it is completely submerged with CSS.
4. Turn on all devices required. Set the temperature of the cooling centrifuge to 4°C. Warm the tube with digestion medium A to 37°C in a heating block. Alternatively, if no heating block is available, warm the medium in a water bath set to 37°C or in a cell culture incubator.
5. Anesthetize a mouse (8–12 weeks old) with isoflurane and euthanize it by cervical dislocation.

**Important:** Animal handling and all associated methods must comply with the appropriate national and institutional regulations.

6. Place the animal in supine position and sterilize the abdominal skin with 70% EtOH. Lift the skin with small forceps and open the abdominal cavity with surgical scissors to expose the digestive tract.
7. Lift the ileum and disconnect the mesentery from the abdominal wall using spring scissors. Remove the intestine by disconnecting it from the stomach and the anus. Immediately place the tissue in a 90-mm culture dish filled with ice-cold oxygenated CSS (Figure 1C-a).

**Important:** To avoid damaging the tissue, do not pull on the mesentery.

8. Use fine forceps and spring scissors to carefully unravel the intestine and remove the mesentery (Figure 1C-b). Be careful not to damage the longitudinal muscle layer.
9. Cut the small intestine including duodenum, jejunum, and ileum into pieces of approximately 5 cm length and store them in the beaker labeled “Dirty.” Remove fecal content by flushing each piece three times with ice-cold CSS using a fire-polished Pasteur pipette, as shown in Figure 1C-c. The use of fire-polished Pasteur pipettes minimizes the risk of tissue ruptures. Collect the fecal content in a separate 50 ml conical tube and store cleaned intestinal pieces in the beaker labeled “Clean.” Cecum and colon are not used during this procedure and are thus discarded.

**Comment:** Although only the small intestine is used in this protocol, the method can also be applied to the colon without further modifications.

10. Cut cleaned intestinal pieces into shorter segments of approximately 2–3 cm length. This length is optimal for further handling of the tissue pieces in following steps.
11. Slide a cleaned intestinal segment onto the tip of a fire-polished Pasteur pipette and secure the tissue with the index finger to prevent it from slipping (Figure 1C-d). Scratch the intestinal segment along the mesenterial line using fine forceps as shown in Figures 1C-e, f to create a gap in the longitudinal muscle layer. Be careful not to damage the underlying tissue layers.
12. Use a cotton swab wetted with CSS to detach the LMMP from the underlying circular muscle layer. Starting at the mesenterial line, gently rub the cotton swab across the tissue with light strokes perpendicular to the longitudinal axis (Figure 1C-g). First, loosen the LMMP along the length of the mesenterial line and then work around the intestinal tube until the LMMP comes off (Figure 1C-h).
13. Immediately transfer the LMMP piece to the cell strainer placed in the “LMMP” beaker (Figure 1C-i). Collecting the tissue in the cell strainer prevents mechanical agitation while oxygenating the CSS. Repeat steps 3.3–12 to 3.3–13 for all intestinal segments.
14. After the LMMPs have been collected, wash the tissue twice with CSS to remove possible contaminations. Using surgical scissors, cut LMMPs into smaller pieces approximately 3–5 mm in length and transfer them into a 1.5 ml centrifuge tube filled with ice-cold oxygenated CSS. Invert the tube 3 times and centrifuge for 1 min at 250 × *g* in a cooling centrifuge set to 4°C.

**Comment:** If desired, some of the cleaned LMMP pieces can be permeabilized and further processed to perform immunohistochemical studies.

### 3.4 Digestion of LMMPs and enrichment of ganglia (timing 6.5 h)

1. Transfer LMMP pieces to the tube containing prewarmed digestion medium A, invert the tube 5 times and incubate for 5 h ± 30 min at 37°C in a cell culture incubator. During the incubation period, cautiously invert the tube 3–4 times every 60 min to facilitate digestion.

**Critical:** Do not vortex the tube and avoid using a nutating mixer during this step, as continuous mechanical agitation can break the myenteric plexus into individual ganglia which are difficult to enrich in later steps of the protocol.

2. Use the incubation period to clean the surgery area. Warm digestion medium B and RPMI medium to 37°C about 1 h before proceeding to the next step.
3. After 5 h, check the progress of the digestion. Cut the first 3 mm of a 1,000 μl pipette tip to increase the tip diameter and use it to transfer 500 μl of the digested LMMPs to a 35 mm culture dish. Pipette slowly to prevent disintegration of myenteric plexus networks. Place the dish under a stereo microscope to evaluate the digestion process. Digestion is complete when the muscle tissue has almost completely disintegrated and the ganglionated network of the myenteric plexus is clearly visible (Figure 1C-j).

**Comment:** If the digestion process is considered incomplete, the material can be transferred back to the tube and incubated for additional 30 min.

4. After completion of the digestion, gently pour the entire suspension containing the myenteric plexus networks into a 35 mm culture dish and add 2 ml of FCS-free DMEM/F12 containing 1% antibiotics to increase visibility of the plexus structures.

**Critical:** During this step, gentle handling of the sample is essential to prevent disintegration of the plexus networks.

5. Allow plexus networks to settle on the bottom of the dish before collecting them under microscopic control with a 200 μl pipette (Figure 1C-k) and transfer them to a fresh 1.5 ml microcentrifuge tube.
6. When all network pieces are collected, fill the tube to 1.5 ml with FCS-free DMEM/F12 containing 1% antibiotics and centrifuge at RT for 1 min at 250 × *g*. Carefully remove the supernatant which contains debris and many muscle cells.
7. Add prewarmed digestion medium B to the plexus networks and incubate for 30 min at 37°C on a nutating mixer placed inside a cell culture incubator to facilitate disintegration of ganglia and release of neurons. Alternatively, use a shaking water bath set to 37°C.
8. After incubation, centrifuge the tube at RT for 1 min at 250 × *g*. Remove and discard the supernatant and add 500 μl of trituration medium to stop the digestion process.
9. Set a 1,000 μl pipette to 400 μl and gently triturate ganglia with 10–15 strokes performed with uniform pressure and speed until the suspension becomes cloudy. Avoid air bubbles while triturating.
10. After trituration, pellet the cells at RT in a centrifuge for 2 min at 250 × *g*. Remove and discard the supernatant. If the cells are to be used without further manipulations, proceed to the next step (3.4–11). If the cells are to be transfected with plasmid DNA, proceed to step 3.5–1 of the protocol.
11. Wash cells twice in complete culture medium to remove most of the debris generated during previous steps. To do this, resuspend the cells in 500 μl of complete culture medium, centrifuge at RT for 2 min at 250 × *g*, discard the supernatant, and repeat once. Then, resuspend washed cells in 300 μl complete culture medium and seed aliquots of 50 μl onto each of the prepared coverslips (Figure 1C-l). Incubate the cells for at least 24 h at 37°C in a humidified incubator with a 95% O_2_ − 5% CO_2_ atmosphere, before performing electrophysiological recordings or any other downstream assay.

**Comment:** Low seeding densities (<10% confluence) are preferred for electrophysiological applications. If other downstream assays are to be used (e.g., immunochemistry), seeding density may need to be adjusted.

### 3.5 Transfection of myenteric neurons with plasmid DNA (timing: 20 min)

1. Resuspend cells in 600 μl of Ca^2+^/Mg^2+^-free PBS and transfer 300 μl of the cell suspension to a fresh 1.5 ml tube to yield two equal lots.

**Comment:** In the following steps, the neurons contained in one tube will be transfected with plasmid DNA encoding the target protein (e.g., Na_V_1.9-L396P channels), while the neurons contained in the second tube will serve as controls, i.e., they are transfected with a control plasmid (e.g., a plasmid encoding Na_V_1.9 wild type channels). Use only transfection-grade endotoxin-free plasmid DNA for transfection.

2. Prepare two fresh 1.5 ml tubes, each containing 16.4 μl of Nucleofector Solution P3 and 3.6 μl of Supplement 1, both included in the P3 Nucleofection kit. Add 2.5 μg plasmid DNA encoding the protein of interest (e.g., Na_V_1.9-L396P) to one tube and 2.5 μg of the control plasmid (e.g., Na_V_1.9 wild type) to the second tube and mix using a pipette.

**Important:** This protocol refers to reporter plasmids that express both the target (Na_V_1.9 or Na_V_1.9-L396P) and an eGFP reporter, eliminating the need for cotransfection. However, the procedure is also compatible with cotransfections. In such a scenario, it is recommended to transfect neurons with a DNA mixture containing target and reporter plasmids at a ratio of 5:1, with no more than 3 μg of total DNA to reduce cell toxicity.

**FIGURE 2:**
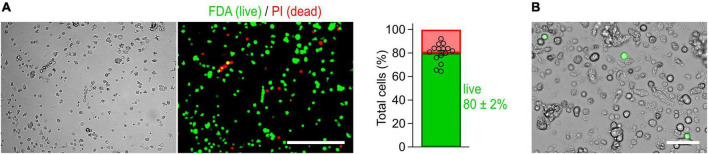
Cells isolated from the myenteric plexus of adult mice are viable. **(A)** Imaging-based viability assay performed with fluorescein diacetate (FDA) and propidium iodide (PI) to evaluate the viability of isolated cells. Cells obtained from myenteric plexus were imaged in brightfield (left) and corresponding fluorescence channels (right, Scale bar: 200 μm) immediately after isolation. Exclusively viable cells are able to convert non-fluorescent FDA into its green fluorescent metabolite fluorescein (green). Damaged cells allowing PI to pass through their membranes are shown in red. *Bar graph*: 80 ± 2% of the isolated cells were viable (cell counting based on 15 image sequences obtained from two independent preparations). **(B)** Live-cell image combining brightfield and fluorescent channels, obtained from a culture of myenteric neurons 40 h post-transfection with a cDNA expression construct encoding Na_V_1.9-L396P mutant channels and enhanced green fluorescent protein (green). Successfully transfected neurons are identified by their bright green fluorescence. Scale bar: 50 μm.

3. Centrifuge both tubes containing the cell suspensions at RT for 1–2 min at 250 × *g*. Remove and discard the supernatant. To each cell pellet add one of the two DNA mixes and resuspend cells by gently pipetting up and down.
4. Transfer each cell suspension into one well of the microcuvette included in the Nucleofector kit. Make sure the bottom of the wells is completely covered with cell suspension and avoid air bubbles while pipetting. Close the cuvette with the lid provided.
5. Place the cuvette into the 4D Nucleofector and apply program CA137 to all wells of the cuvette filled with cell suspension.
6. After transfection, remove the cuvette from the 4D Nucleofector device and immediately add 150 μl of prewarmed RPMI Medium to each well containing transfected cells. Gently mix cell suspensions using a pipette. Place the cuvette for 10 min at 37°C in a humidified 95% O_2_ − 5% CO_2_ incubator to allow the cells to recover from transfection.
7. Seed 50–60 μl of cell suspension per coverslip and incubate cells at 37°C in a humidified 95% O_2_ − 5% CO_2_ incubator.
8. Verify cell viability 24–48 h post-transfection. Successfully transfected cells can be identified visually by their bright green eGFP-mediated fluorescence (Figure 2B). Patch-clamp recordings of transfected neurons can be performed 24–72 h after seeding.

## 4 Results

**FIGURE 3:**
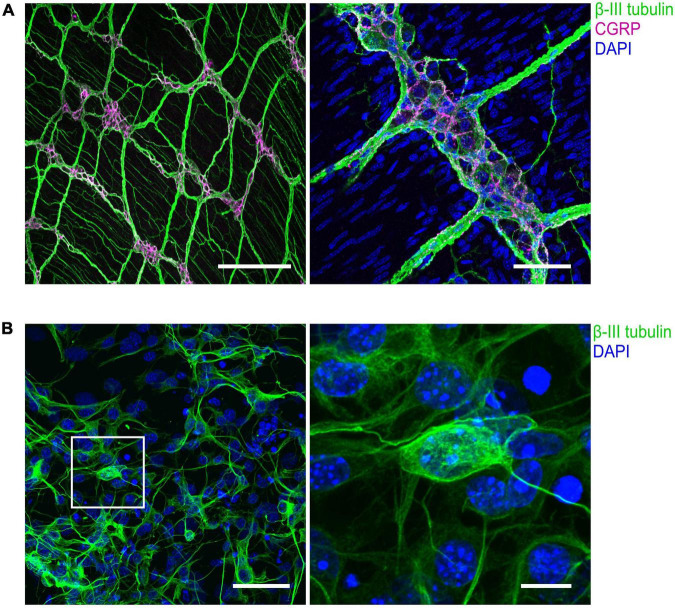
Immunohistochemical visualization of enteric neurons isolated from mouse longitudinal muscle/myenteric plexus (LMMP) preparations. **(A)** Representative confocal images of whole-mount LMMP samples showing the ganglionated myenteric plexus which is strongly attached to the longitudinal muscle layer. Neuronal fibers exhibit immunoreactivity for the pan-neuronal marker β-III-tubulin (green). Structures immunoreactive for the neuromodulator calcitonin gene-related peptide (CGRP) are shown in magenta. Nuclei were stained with DAPI (blue, right). In the left image, staining of nuclei was omitted. Scale bars: 200 μm (left) and 50 μm (right). **(B)** (Left) Culture of cells isolated from myenteric plexus. Time in culture was 64 h. Neurons were marked with an antibody against β-III-tubulin (green), nuclei were counterstained with DAPI (blue). (Right) Enlarged view. Scale bars: 50 μm (left) and 10 μm (right).

The method has been optimized to isolate, transfect, and cultivate myenteric neurons for electrophysiological applications. First, the longitudinal muscle layer with adherent myenteric plexus (Figures 1C, 3A) was detached from the intestinal tube and then subjected to a two-step enzymatic treatment. Structurally intact plexus networks were isolated using Liberase, a highly purified collagenase formulation causing selective disintegration of the collagen-containing muscle tissue (Figure 1C-j). Intact plexus pieces were harvested (Figure 1C-k) and subjected to a second enzymatic digestion to release the neurons from pre-purified ganglia. We found that this approach is robust and reproducibly yields mixed cultures enriched with living myenteric neurons (Figures 1C-l, 2B). In our hands, the overall viability of isolated cells is approximately 80% (Figure 2A).

Immediately after isolation, the majority of cells exhibit a round morphology (Figure 1C-l), making the identification of individual neurons difficult at this time point. However, after 48–72 h, neurites become visible allowing visual identification of neurons (Figure 3B). After 24 h in culture, the cells are well attached to the coverslips and can be transferred to an experimental chamber for functional studies. Possible pitfalls and troubleshooting advice can be found in Table 2.

**TABLE 2:** Possible pitfalls and troubleshooting.

Step	Issue	Countermeasures
3.3–12	LMMP does not separate from the intestinal segment.	Scratching with forceps along the mesenterial line must be performed carefully to cut through the longitudinal muscle while leaving the circular muscle intact. Apply light, uniform pressure while rubbing the tip of the forceps in one stroke along the mesenterial line.
3.4–3	After digestion with digestion medium A, only few plexus networks are freed from muscle tissue.	Increase incubation time or enzyme concentration. Liberase allows extended incubation times without risking over digestion (Grundmann et al., 2015).
3.4–11	After seeding cells, many large fragments of undigested tissue are visible.	Mechanical stress by trituration may have been insufficient to release the neurons from the ganglia. Extend the incubation time with digestion medium B (step 3.4–7) and increase the number of strokes, speed and pressure when triturating (step 3.4–9).
3.4–11	The cell culture is contaminated.	Gently wash the LMMP segments at least twice with CSS solution (step 3.3–14) before proceeding to the first digestion step. If necessary, include additional washing steps and increase the concentration of antibiotics in the growth medium.
3.4–11	Low cell viability.	Cell viability depends on the quality of the isolated LMMP pieces and also on the age of the animals used. Keep the temperature of the intestinal tissue low until the first enzymatic treatment by dissecting the LMMP tissue in ice-cold CSS. Reduce the time to harvest LMMPs and use younger animals that typically yield more robust cells.
3.5–8	Cells are not transfected.	The amount and viability of isolated cells, the intensity of the electrical pulses used for electroporation, and the concentration of DNA in the transfection mix are all factors that affect transfection efficiency. Increase the number of cells in the transfection mix by combining the cells from two animals. Use an alternate program of the electroporation device that delivers stronger pulses. Increase the amount of DNA in the transfection mix, but do not exceed 3 μg of total DNA per micro-cuvette to prevent cell toxicity.

**FIGURE 4:**
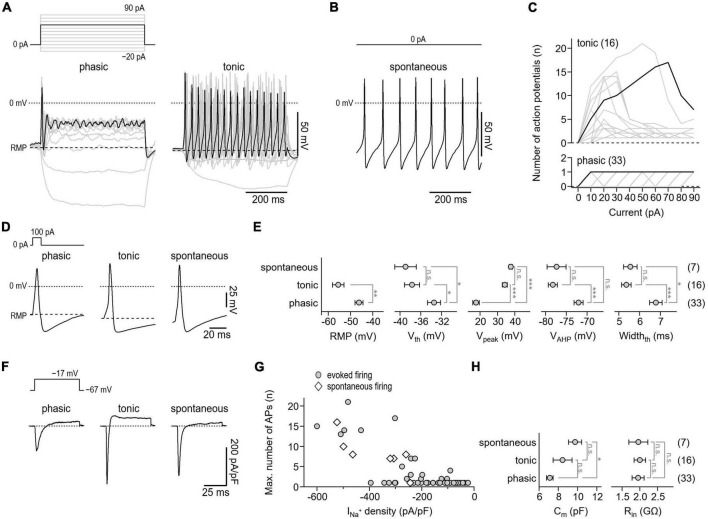
Action potential characteristics of isolated murine enteric neurons. **(A)** Representative voltage responses (bottom) of cultivated enteric neurons to 500-ms current injections increasing from –20 to 90 pA in steps of 10 pA (top). Of a total of 56 neurons analyzed, phasic activity was observed in 33 cells (phasic, left) while 16 cells showed tonic firing (tonic, right). The 60 pA stimulation step (top) and corresponding voltage responses (bottom) are highlighted in black. **(B)** Representative train of action potentials recorded from a spontaneously firing enteric neuron (bottom) at zero current injection (top). Spontaneous activity was observed in 7 of 56 enteric neurons. **(C)** Number of action potentials plotted as a function of injected current for phasically (bottom) and tonically (top) active neurons. Data in black correspond to cells shown in panel **(A)**. **(D)** Representative single action potentials (bottom) in response to brief 10-ms depolarizing current injections of 100 pA (top) recorded from neurons showing phasic, tonic, or spontaneous activity. **(E)** Parameters characterizing the shape of individual action potentials obtained from experiments as shown in panel **(D)**. RMP, resting membrane potential; *V*_th_, action potential voltage threshold; *V*_peak_, action potential peak voltage; *V*_AHP_, minimum voltage of action potential afterhyperpolarization; *Width*_th_, action potential width at the threshold voltage *V*_th_. **(F)** Representative current responses of enteric neurons showing phasic, tonic, or spontaneous activity (bottom) recorded with voltage pulses to –17 mV to monitor Na_*V*_-mediated inward currents. Measurements were obtained from the same cells as shown in panels **(A,B)**, the holding potential was –67 mV (top). **(G)** Maximum number of repetitively fired action potentials obtained in experiments as shown in panels **(A,B)** as a function of inward current density as shown in panel **(F)**, demonstrating that repetitive firing scales with the expression of Na_V_ channels. **(H)** Cell membrane capacitance (C_m_) and input resistance (R_in_) of phasically, tonically, and spontaneously firing neurons. Data points in panels **(E,H)** are means ± s.e.m., with the numbers of cells analyzed provided in parentheses. Statistical significance between pairs of data was tested with a two-sided Student’s *t*-test: ***P < 0.001, **P < 0.01, *P < 0.05, n.s., not significant.

Following cultivation for 24–72 h, the electrical activity of the neurons was assessed in whole-cell patch-clamp experiments. Since the ability to fire action potentials is a defining feature of neurons, we first analyzed the action potential firing characteristics of the cells under current-clamp conditions. To trigger action potential firing, cells were stimulated with 500 ms current injections ranging from −20 to 90 pA. Of a total of 56 neurons tested, 33 neurons responded to this stimulation paradigm with no more than one action potential per stimulus (phasic activity), whereas 16 neurons responded with repetitive action potentials depending on the stimulation intensity (tonic activity) (Figures 4A, C). In addition, 7 neurons generated action potentials spontaneously, i.e., in the absence of any current stimulus (Figure 4B). This is consistent with previous *in vitro* studies that have demonstrated tonic, phasic, and spontaneous firing patterns in both embryonic and adult murine myenteric neurons (Hao et al., 2011, 2012, 2013).

To facilitate comparison of action potential waveforms between the three groups, individual action potentials were triggered with brief 10 ms current injections. As shown in Figures 4D, E, the parameters characterizing the shape of action potentials, that is, the voltage threshold for action potential firing (*V*_*th*_), the action potential peak voltage (*V*_*peak*_), the action potential after hyperpolarization voltage (*V*_*AHP*_), and the action potential duration (*Width*_*th*_) were indistinguishable between tonically and spontaneously active neurons. Compared with these two groups, action potentials of phasically active neurons were characterized by a lower *V*_*peak*_ (phasic: 17.6 ± 1.9 mV; tonic: 34.3 ± 1.5 mV; spontaneous: 37.8 ± 1.0 mV), a higher *V*_*th*_ (phasic: −33.4 ± 1.3 mV; tonic: −37.7 ± 1.4 mV; spontaneous: −38.9 ± 2.1 mV), a depolarized *V*_*AHP*_ (phasic: −72.1 ± 1.1 mV; tonic: −78.3 ± 1.1 mV; spontaneous: −77.4 ± 2.3 mV), and by a longer duration (phasic: 6.8 ± 0.3 mV; tonic: 5.4 ± 0.2 mV; spontaneous: 5.6 ± 0.3 mV). In addition, the resting membrane potential (RMP) of phasically active neurons was depolarized by about 9 mV compared with that of tonically active neurons (phasic: −46.1 ± 1.8 mV; tonic: −55.5 ± 2.5 mV) (Figure 4E). As expected, all neurons tested expressed functional Na_*V*_ channels, albeit to varying degrees. Moreover, as shown in Figures 4F, G, the Na_*V*_-associated current density correlated with the action potential phenotype of the cells. Passive membrane properties such as membrane capacitance (*C*_*m*_) and cell input resistance (*R*_*in*_) varied only slightly or were indistinguishable among the three groups (Figure 4H).

Myenteric neurons express a heterogenous population of Na_*V*_ subtypes that can be divided in two groups based on their sensitivity to the neurotoxin tetrodotoxin (TTX). At least four TTX-sensitive (TTXs, IC_50_ ≤ 10 nM: Na_*V*_1.1, Na_*V*_1.3, Na_*V*_1.6, Na_*V*_1.7) and two TTX-resistant (TTXr, IC_50_ ≥ 1 μM: Na_*V*_1.5, Na_*V*_1.9) Na_*V*_ isoforms have been identified in the ENS of mice (Hao et al., 2012; Hirst et al., 2015).

**FIGURE 5:**
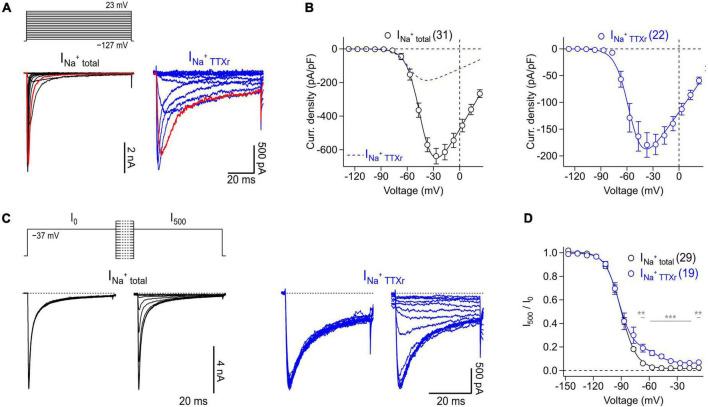
Voltage-clamp recordings of Na_V_ channels endogenously expressed in murine enteric neurons. **(A)** Families of current traces obtained from representative enteric neurons in the absence (I_Na_^+^ total, black) or presence (I_Na_^+^ TTXr, blue) of 1 μM TTX in response to a series of depolarizing test pulses (top) increasing from –127 to 23 mV in steps of 10 mV. Traces shown in red correspond to a test pulse voltage of –37 mV. **(B)** Peak current densities (open circles) recorded in the absence (I_Na_^+^ total, black) or presence (I_Na_^+^ TTXr, blue) of 1 μM TTX as a function of test pulse voltage, obtained from experiments as shown in panel **(A)**. Continuous curves are superimposed fits according to Eq. 1 describing the voltage dependence of activation of Na_V_ channels. To facilitate comparison of both conditions, the data fit to currents in the presence of TTX is shown as blue dashed line in the left panel. **(C)** Families of current traces obtained from representative enteric neurons in the absence (I_Na_^+^ total, black) or presence (I_Na_^+^ TTXr, blue) of 1 μM TTX with test pulses to –37 mV, applied before (I_0_) and after (I_500_) a 500-ms conditioning period at voltages ranging from –147 to –7 mV in steps of 10 mV (top). **(D)** Ratio of peak currents measured before and after 500-ms conditioning (I_500_/I_0_) as a function of conditioning voltage, obtained from experiments as shown in panel **(C)** to characterize the voltage dependence of steady-state inactivation of Na_V_ channels. Superimposed fits are Boltzmann functions according to Eq. 2. In the presence of 1 μM TTX (I_Na_^+^ TTXr, blue), inactivation of Na_V_ channels displayed two components reflecting inactivation properties of different TTXr channel subtypes such as Na_V_1.5 and Na_V_1.9 (Osorio et al., 2014), both of which are endogenously expressed in enteric neurons. In panels **(A–D)** the holding potential was –137 mV. Data points in panels **(B,D)** are means ± s.e.m. with numbers of experiments given in parentheses. Significance between pairs of data was tested with a two-sided Student’s *t*-test: ***P < 0.001, **P < 0.01.

Thus, isolated myenteric neurons were subjected to voltage-clamp recordings to analyze their Na_*V*_-mediated current components both in the absence and presence of TTX (Figure 5). Under control conditions, i.e., without TTX present, depolarizing voltage steps between −127 and 23 mV evoked large, fast activating Na^+^ inward currents (Figure 5A, *left*) that reached a maximum current density of −637.0 ± 46.2 pA/pF at −27 mV (Figure 5B, *left*). Inactivation kinetics of the channels exhibited two components: a major fast inactivating component as it is characteristic for most TTX-sensitive (TTXs) Na_*V*_ subtypes and a minor slow inactivating component most likely mediated by TTX-resistant (TTXr) Na_*V*_ isoforms (Catterall et al., 2005; Lee and Ruben, 2008). Analysis of peak inward currents as a function of test pulse voltage revealed a half-maximal voltage of channel activation, *V*_*m*_, of −43.6 ± 1.6 mV and an associated slope factor reflecting the voltage dependence of channel activation, *k*_*m*_, of 8.0 ± 0.4 mV (Figure 5B, *left*). Adding 1 μM TTX inhibited specifically the fast inactivating current component (Figure 5A, *right*) leading to a block of total Na^+^ current by 70% (−191.6 ± 27.3 pA/pF at −37 mV) and a significant hyperpolarizing shift of *V*_*m*_ by −7.7 mV (−51.3 ± 0.9 mV, *P* < 0.01) (Figure 5B, *right*).

Analysis of the voltage dependence of steady-state channel inactivation, measured after 500 ms conditioning episodes at various voltages in the absence of TTX (Figures 5C, D), revealed a half-maximal voltage of channel inactivation, *V*_*h*_, of −83.4 ± 0.2 mV and an associated slope factor of 8.3 ± 0.2 mV. In the presence of 1 μM TTX, channels inactivated with two components described by a linear combination of components. This is consistent with a previous study demonstrating that TTXr currents of myenteric neurons are mediated exclusively by Na_*V*_1.5 and Na_*V*_1.9 (Osorio et al., 2014). The larger of the two components, accounting for 85% of the TTXr currents, was characterized by a *V*_*h*_ value of −93.1 ± 0.9 mV and an associated value for *k*_*h*_ of 7.7 ± 0.9 mV, most likely reflecting the properties of Na_*V*_1.5. The smaller component (14.1%), mediated by slow inactivating currents, was characterized by a *V*_*h*_ value of −52.4 ± 2.3 and a slope factor (*k*_*h*_) of 8.2 ± 2.1 mV which can be attributed to murine Na_*V*_1.9 channels.

**FIGURE 6:**
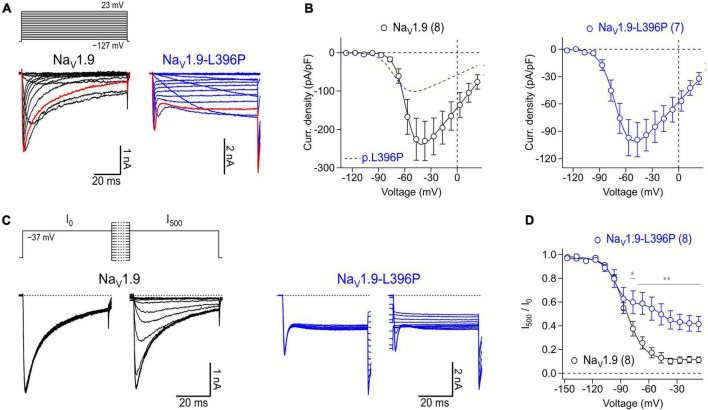
Expression of recombinant human Na_V_1.9 wild type and mutant channels in isolated mouse enteric neurons. **(A)** Representative families of current traces obtained from enteric neurons transfected with cDNA constructs coding for wild type Na_V_1.9 (black) or Na_V_1.9-L396P mutant channels (blue) in response to a series of depolarizing test pulses (top) increasing from –127 to 23 mV in steps of 10 mV. Red traces were recorded at –37 mV illustrating the different inactivation kinetics of wild type and mutant channels. **(B)** Peak current densities (open circles) recorded from neurons transfected with wild type Na_V_1.9 (black) or Na_V_1.9-L396P mutant channels (blue) as a function of test pulse voltage, obtained from experiments as shown in panel **(A)**. Continuous curves are superimposed fits according to Eq. 1 describing the voltage dependence of channel activation. For better comparison, the data fit to currents generated by neurons expressing mutant channels (blue dashed line) is superimposed in the left panel. **(C)** Families of current traces obtained from representative enteric neurons transfected with type Na_V_1.9 (black) or Na_V_1.9-L396P mutant channels (blue) in response to test pulses to –37 mV which were applied before (I_0_) and after (I_500_) a 500-ms conditioning period at voltages ranging from –147 to –7 mV in steps of 10 mV (top). **(D)** Ratio of peak currents measured before and after 500-ms conditioning (I_500_/I_0_) as a function of conditioning voltage, obtained from experiments as shown in panel **(C)** to characterize the voltage dependence of steady-state inactivation of Na_V_ channels. Superimposed fits are Boltzmann functions according to Eq. 2. Note, currents generated by cells expressing Na_V_1.9-L396P mutant channels exhibit a biphasic voltage dependence of inactivation along with a pronounced non-inactivating component. All recordings were obtained in presence of 1 μM TTX, data points in **(B,D)** are means ± s.e.m. with numbers of experiments provided in parentheses. In panel **(D)**, significance between pairs of data was tested with a two-sided Student’s *t*-test: **P < 0.01, *P < 0.05.

To illustrate the suitability of isolated myenteric neurons for overexpression studies, cells were transfected with cDNA constructs encoding either Na_*V*_1.9 wild-type or mutant Na_*V*_1.9-L396P associated with congenital analgesia and severe gastrointestinal dysfunction (King et al., 2017). In the presence of 1 μM TTX, neurons transfected with a cDNA encoding Na_*V*_1.9 wild-type channels gave rise to robust slow inactivating current responses typical for this Na_*V*_ subtype (Figure 6A, *left*), reaching a maximum current density of −230.0 ± 51.5 pA/pF at −37 mV (Figure 6B, *left*). Analysis of the voltage dependence of peak current amplitudes revealed a half-maximal voltage of channel activation of −57.9 ± 0.4 mV and a corresponding slope factor of 6.5 ± 0.3 mV. By contrast, current responses of neurons expressing p.L396P mutant channels exhibited a pronounced non-inactivating component that was not evident in cells expressing the wild type, indicating impaired inactivation of overexpressed mutant channels (Figure 6A, *right*). Furthermore, the maximal current amplitude of neurons expressing mutant channels was reduced (−99.0 ± 18.7 pA/pF at −47 mV, *P* < 0.05), and their voltage-dependent activation was shifted to hyperpolarizing potentials, consistent with a half-maximal activation voltage of −70.4 ± 0.4 (*P* < 0.01) and a slope factor of −8.5 ± 0.3 (n.s.).

Analysis of steady-state inactivation revealed a half-maximal inactivation voltage of −85.3 ± 0.7 mV and a corresponding voltage dependence of 9.8 ± 0.7 mV for neurons transfected with Na_*V*_1.9 wild type. Currents of neurons expressing mutant channels inactivated with two clearly distinguishable components, one resembling inactivation of TTXr channels endogenous to the neurons (*V*_*h*_: of −96.8 ± 1.4 mV, *k*_*h*_: 6.2 ± 1.2 mV) and another resembling the activity of inactivation-deficient p.L396P mutant channels (*V*_*h*_: of −47.7 ± 3.5 mV, *k*_*h*_: 7.0 ± 3.2 mV) (Figures 6C, D). The fraction of non-inactivating mutant channels was estimated to be 0.30 ± 0.05. Collectively, the data demonstrate successful overexpression of recombinant human Na_*V*_1.9 channels in murine myenteric neurons and confirm previous results obtained with transfected murine dorsal root ganglion neurons (King et al., 2017) showing that the p.L396P mutation confers gain-of-function properties to Na_*V*_1.9 by enhancing channel activation and impairing channel inactivation.

## 5 Discussion

Isolated myenteric neurons represent a useful *in vitro* model to study physiological and pathophysiological conditions of the enteric nervous system. The presented cell isolation method yields mixed cell populations enriched with myenteric neurons (Figure 3B). The functional properties of the isolated enteric neurons can be analyzed using electrophysiological assays such as patch-clamp recordings. However, the cells are also compatible with other downstream assays, including immunochemistry, calcium imaging, or single-cell nucleic acid approaches. In combination with modern approaches such as RNA sequencing after patch-clamp (Lipovsek et al., 2021), the method could help to gain insight into the molecular and functional diversity of enteric neuron subtypes (Morarach et al., 2021) to better understand their roles under both physiological and pathophysiological conditions. In addition, isolated myenteric neurons can be transfected with cDNA constructs, allowing systematic overexpression studies, for example, to study gene defects affecting the neurons, as demonstrated here by overexpression of Na_V_1.9-L396P mutant channels which are associated with severe gastrointestinal symptoms in affected patients. The transfection of murine myenteric neurons is a valuable experimental option as it allows to study the function of target proteins such as p.L396P mutant channels in a disease-related physiological context. This is particularly important because experimental access to primary human enteric neurons is often limited for ethical and practical reasons.

This protocol has been optimized to isolate myenteric neurons from C57BL/6JRj mice. However, the method can be easily adapted to other mouse strains including genetically modified strains or other small-sized animals further increasing potential applications. Here, we restricted the isolation of myenteric neurons to the small intestine of mice because of the large tissue volume available and the comparatively easy separation of the longitudinal muscle from the underlying circular muscle layer. But with minimal adaptations, the procedure can also be used to obtain neurons from colon.

### 5.1 Advantages of the method

A major difference between our protocol and other methods used for isolation of enteric neurons (Hao et al., 2012; Smith et al., 2013) is that it combines a particularly gentle two-step enzymatic treatment regime with steps to efficiently enrich myenteric neurons. First, intact myenteric plexus networks are obtained from isolated LMMP pieces by selectively disintegrating the muscle tissue. In a subsequent step, the pre-purified plexus pieces are enzymatically dissociated into individual neurons. By using highly purified enzymes with minimal batch-to-batch variation, the method allows for extended digestion times of several hours. This minimizes the amount of undigested tissue in the preparation, resulting in neuron-enriched cultures with reproducibly high proportions of viable cells (Figures 2A, 3B). Neurons isolated with this method can be cultivated and functionally analyzed for several days.

### 5.2 Limitations of the method

Potential limitations of the method include the lack of intact neuronal circuits in the final cell cultures, the exclusion of neurons from mucosal and submucosal tissue layers, and the low yield of neurons relative to the volume of tissue used for isolation. Furthermore, it is possible that not all subtypes of enteric neurons are preserved in the final cell cultures. In addition, the culture conditions could trigger adaptive processes in the neurons that alter their physiological profiles, as has been described for other primary neuron culture models, for example, dorsal root ganglion neurons (Black et al., 1997; Fjell et al., 1999; Leffler et al., 2002).

### 5.3 Throughput and time requirements

With some practice, an experienced experimenter can dissect and process the small intestine of up to two mice within 8 h (Figure 1A), obtaining enough cells to prepare 12 coverslips with low to medium cell density. If cells from more than two animals are needed for downstream experiments, we recommend a second person assisting with the dissection to keep the preparation time short, which is essential for obtaining viable neurons.

## Data availability statement

The original contributions presented in this study are included in the article/supplementary material, further inquiries can be directed to the corresponding author.

## Author contributions

SK, LT, A-KH, CS, and NH performed the experiments. SK, NH, and EL analyzed the data. SK, NH, IK, RB, CN, and EL designed the research and wrote the manuscript with input from co-authors. All authors contributed to the article and approved the submitted version.
